# Plasma proteomic signatures predict incident benign prostatic hyperplasia: a prospective cohort study of 20 996 men

**DOI:** 10.7189/jogh.16.04185

**Published:** 2026-06-12

**Authors:** Hao Li, Yangchang Zhang, Li Chen, Jiuhong Yuan, Feng Qin, Xianding Wang, Yang Xiong

**Affiliations:** 1 Division of Internal Medicine, Institute of Integrated Traditional Chinese and Western Medicine, Regenerative Medicine Research Center, West China Hospital, Sichuan University, Chengdu, China; 2 Department of Rheumatology and Immunology, The First Affiliated Hospital of Chongqing University of Chinese Medicine, Chongqing, China; 3 Institute of Child and Adolescent Health, School of Public Health, Peking University, Beijing, China; 4 Department of Urology, Institute of Urology, Kidney Transplantation Center, West China Hospital, Sichuan University, Chengdu, China

## Abstract

**Background:**

Benign prostatic hyperplasia (BPH) is a prevalent disease in elderly men. However, the plasma proteomic signatures for incident BPH are absent, hindering early prediction and risk stratification. We aimed to identify plasma proteins associated with incident BPH and the implicated biological pathways.

**Methods:**

This prospective cohort was constructed based on the UK Biobank, enrolling 20 996 BPH-free males at baseline. We used the Olink Explore platform to determine the abundances of 2920 plasma proteins. We used Cox regression models to evaluate associations and the Extreme Gradient Boosting model to perform feature selection and predictive modelling. We performed pathway enrichment and Mendelian randomisation analyses to elucidate the molecular pathways involved and assess causality.

**Results:**

During the median follow-up period of 13.38 years, 2405 incident BPH cases were recorded. Cox regression identified 92 plasma proteins significantly associated with BPH risk (false discovery rate <0.05). Extreme Gradient Boosting model prioritised a three-protein signature: TSPAN1 (hazard ratio (HR) = 1.24) and KLK3 (HR = 1.34) as risk factors, and EDA2R (HR = 0.83) as a protective factor. This three-protein panel achieved an AUC of 0.71 (95% confidence interval = 0.69–0.73) for predicting BPH onset. Enrichment analysis revealed the associated proteins were mainly involved in immune-inflammatory pathways and stromal remodelling. As revealed by Mendelian randomisation, TSPAN1 and KLK3 were causally associated with incident BPH.

**Conclusions:**

We identified a three-protein panel (TSPAN1, KLK3, EDA2R) which can predict incident BPH. The findings highlight potential targets for non-invasive risk assessment and therapy.

Benign prostatic hyperplasia (BPH) is a common disease in elderly men. It often causes lower urinary tract symptoms and adversely impacts patients' quality of life [1]. The global burden of BPH affects up to 79 million men aged >60 years [2], but the clinical management is largely reactive, depending on the presentation of symptoms. This clinical need highlights the lack of biomarkers for risk stratification. The discovery of such markers would enable a shift toward earlier, more individualised monitoring and pre-symptomatic treatment.

In this setting, high-throughput plasma proteomics has recently emerged as a tool for the multiplexed quantification of thousands of circulating proteins and offers a non-invasive window into systemic physiology and disease-relevant pathways [3]. Proteomic signatures have proven highly valuable for risk prediction in complex disease states, such as prostate cancer [4] and cardiometabolic diseases [5], and thus hold promise for revealing the biology and enhancing pre-clinical detection of BPH.

The utility of plasma biomarkers to delineate the aetiology of BPH and risk stratification is limited. Although routinely measured, serum prostate-specific antigen (PSA) (encoded by KLK3) is an indicator of general prostate activity, rather than a specific predictor of BPH. PSA is often confounded by inflammation and other conditions [6]. The role of other prostate-derived proteins, such as microseminoprotein-beta (MSMB) has been explored mainly in the context of prostate cancer [7] with limited work to delineate the role in BPH pathogenesis. These studies highlight the absence of large prospective studies that systematically screen the plasma proteome to identify protein signatures that predict future incidence of BPH. In addition, the systemic biological pathways captured by these circulating biomarkers are not well understood.

To address this concern, we performed a prospective proteome-wide association study of 2920 plasma proteins from the UK Biobank (UKB). We aimed to discover and validate protein biomarkers for incident BPH, assess their predictive performance by machine learning, and investigate the involved biological pathways. Through the identified proteomic signature, novel predictive tools for early risk assessment can be developed, facilitating clinical decision-making.

## METHODS

We analysed the data from UKB [8]. We adhered to the Journal of Global Health’s GRABDROP guidelines (Table S1 in the **Online Supplementary Document**) [9].

### Study design and participants

We performed a prospective cohort analysis using the UKB [8], a large-scale biomedical database of >500 000 participants recruited between 2006 and 2010 from the UK. The UKB includes a comprehensive baseline assessment of physical measurements, biological samples, and detailed health and lifestyle information. The participants were followed up by electronic health records. The cohort started with 53 014 UKB participants with available plasma proteomic data. After sequentially excluding 4286 participants with >50% missing plasma protein data, 1404 participants with a prevalent diagnosis of BPH at baseline and 26 328 female participants, we obtained a final cohort of 20 996 male participants who were followed from baseline until the first BPH diagnosis or censored at the end of follow-up (*i.e.* 1 August 2023). During follow-up, 2405 incident BPH cases were recorded, with 18 591 participants remaining BPH-free (Figure S1 in the **Online Supplementary Document**).

### Measurement of plasma protein abundance

We quantified plasma protein levels using the Olink Explore 3072 platform, an antibody-based proximity extension assay. This platform measured 2941 protein analytes representing 2923 unique proteins [10]. We performed the assay on plasma samples from 54 219 UKB participants, providing normalised protein expression values for protein analytes. As part of rigorous quality control, we excluded protein analytes with missing values >50% of the cohort from subsequent analysis and kept 2920 unique proteins, with no duplicate assays or control probes. For the remaining proteins with a lower proportion of missing data (<50%), we imputed missing plasma protein levels using the mean (x̄) of the respective protein. This processed data set formed the basis for all downstream analyses examining the association between circulating protein abundance and incident BPH.

### Diagnosis of BPH and included covariates

The diagnosis of incident BPH was the primary outcome of this study. We identified cases using the International Classification of Diseases, Tenth Revision code N40, sourced from linked hospital inpatient records within the UKB [11]. To ensure a focus on new-onset disease, we excluded participants with a recorded diagnosis of BPH before or at the baseline assessment.

We included a range of covariates, measured at baseline, in the analysis to account for potential confounding. We selected these variables based on established associations with BPH risk and proteomic profiles. Sociodemographic factors included ethnicity (white/non-white), family income (<GBP 31 000 and ≥ GBP 31 000), and educational levels (college or above, high school or equivalent, and less than high school). Lifestyle factors comprised smoking status (never, previous, current), alcohol consumption frequency (never, previous, current), and body mass index (<25, 25–30, and >30 kg/m^2^). Clinical and biochemical factors included the use of cholesterol-lowering drugs, the use of blood-pressure-lowering drugs, as well as circulating levels of glycated haemoglobin (HbA1c, mmol/mol), and low-density lipoprotein (LDL, mmol/L) cholesterol. Data for these covariates were collected through touchscreen questionnaires, verbal interviews, physical measurements, and biomarker assays during the initial UKB assessment visit.

### Polygenic Risk Score (PRS) stratification in UKB

To account for the genetic predisposition to BPH, we constructed the PRS for each participant. The PRS was constructed using genetic data from UKB and summary statistics from a large-scale genome-wide association study of BPH in the FinnGen R12 release (41 137 cases and 153 573 controls) [12]. We selected 55 independent single-nucleotide polymorphisms associated with BPH at a genome-wide significance threshold (*P* < 5 × 10^−8^, linkage disequilibrium *r^2^*<0.001 within a window of 10 000 kb) (Table S2 in the **Online Supplementary Document**). We computed individual PRSs using PLINK v1.9 [13] by summing the dosage of each risk allele weighted by its effect size from the FinnGen BPH GWAS. To facilitate analysis, we stratified the participants into high and low genetic risk groups based on the median value of the PRS distribution within the cohort. This PRS variable was included in subsequent models to validate the association between plasma proteins and BPH.

### Statistical analyses

#### Observational association of plasma proteins with incident BPH

We summarised the baseline characteristics of the study participants, stratified by incident BPH status, as x̄ with standard deviations (SDs) for continuous variables and as numbers and percentages for categorical variables. We assessed group differences using the *t* test or the χ^2^ test, as appropriate. We imputed missing values in the covariates using the x̄ value for continuous variables and the mode for categorical variables.

We assessed the association between each plasma protein and the risk of incident BPH using Cox proportional hazards models. We set the time scale as the age of BPH onset. We constructed two models: a minimally adjusted model controlling for family income, body mass index, ethnicity, and educational levels (model 1), and a fully adjusted model additionally including smoking status, alcohol consumption, cholesterol-lowering drugs, blood-pressure-lowering drugs, HbA1c, and LDL (model 2). We presented the results as hazard ratios (HRs) with 95% confidence intervals (CIs) per SD increase in protein abundance. To account for multiple testing, we applied a false discovery rate (FDR) correction across all tested proteins, considering proteins with an FDR-adjusted *P*-value <0.05 in model 1 and model 2 significantly associated with BPH incidence. We estimated the associations of proteins with PRS for BPH using linear regression adjusted for the same covariates in model 2. To explore potential effect modification, we performed subgroup analyses. We stratified the participants by median age (<58 *vs.* ≥58 years) and by median PRS (low *vs.* high genetic risk). We examined the associations of significant proteins with BPH risk within each stratum. We reported the results according to the STROCSS 2025 guidelines [14].

#### Causal effects of identified proteins on BPH

To elucidate the functional relationships and potential biological mechanisms linking the identified plasma proteins to BPH pathogenesis, we performed protein-protein interaction (PPI) network, GO/KEGG, and Reactome enrichment analysis based on the significant proteins in a previous protein-wide association study. We constructed the PPI network using the STRING database, version 12.0 [15], with the species set to Homo sapiens. We retained only physical and functional interactions with a combined confidence score >0.9 to ensure high reliability. Using a stringent STRING confidence threshold of >0.9, only 22 of the 92 BPH-associated proteins formed a single connected PPI network. This high threshold ensures a very low false positive rate but inevitably excludes weaker or less documented interactions. Therefore, the remaining proteins may still participate in BPH pathogenesis through independent or database-incomplete pathways. In addition, we used k-means clustering with a pre-defined number of eight clusters according to the number of networks to find functional clusters. Subsequently, we employed the Enrichr web server [16] to perform GO/KEGG and Reactome enrichment analysis. We considered enriched terms with *P* < 0.05 statistically significant. We visualised the most relevant enriched terms using bar plots to summarise the key biological themes implicated by the proteomic signature of BPH risk.

To evaluate potential causal relationships between the identified plasma proteins and BPH risk, we performed two-sample cis-Mendelian randomisation (MR) analyses. We restricted these analyses to the significantly risk proteins identified in the primary proteomic screen. Genetic instrument for each protein was the index cis-protein quantitative trait locus (cis-pQTL), defined as the most strongly associated variant within 500 kb of the encoding gene that reached genome-wide significance (*P* < 5 × 10^−8^). These cis-pQTLs were detected in 35 571 UKB-Pharma Proteomics Project participants with European ancestry [10]. We sourced summary statistics for BPH from the FinnGen R12 release. We calculated the causal estimate for each protein using the Wald ratio method. We considered a nominal *P*-value <0.05 statistically significant. We considered proteins displaying significant MR estimates with an effect direction consistent with the observational findings from the Cox regression models to support a causal role in BPH pathogenesis. These MR analyses provide supportive, rather than definitive, evidence for causal relationships between plasma proteins and incident BPH. These findings should be interpreted cautiously due to the inherent limitations of single-variant cis-pQTL instruments, including potential weak instrument bias and horizontal pleiotropy.

#### Constructing plasma protein biomarker prediction models

To develop the best predictive model for incident BPH risk based on plasma proteomic signatures, we employed the Extreme Gradient Boosting (XGBoost) algorithm. We first randomly assigned 70% of participants to a training set and 30% to a hold-out test set. Within the training set, an XGBoost model was initially trained using all significant plasma proteins associated with incident BPH. To identify a concise and high-performance biomarker panel, we implemented a sequential forward selection procedure. We iteratively added proteins to the model in descending order of their importance scores derived from the initial model. We evaluated the performance of each progressively larger panel using 5-fold cross-validation within the training set, with the area under the receiver operating characteristic curve (AUC) as the primary metric. The selection process identified a refined panel of three leading proteins, TSPAN1, KLK3, and EDA2R, that together yielded the best predictive performance. We interpreted the contribution of each protein feature to the model's predictions using SHapley Additive exPlanations (SHAP) values, which quantify the marginal impact of each feature on the individual predicted risk scores. We performed all feature selection steps, including XGBoost-based ranking and forward selection, strictly within the training set using cross-validation. We did not access the hold-out test set during any stage of feature selection or model training, and we used these data solely for the final evaluation of the best model.

We further assessed the final predictive performance based on the three-protein panel in the independent test set. We evaluated and compared three distinct models: a plasma protein model built with the identified three-protein panel; a clinical model built with baseline covariates in model 2; and a combined model integrating both the three-protein panel and clinical covariates in model 2. In Cox models used for prediction, we treated the age of BPH onset as the time scale and thus did not include it as a covariate in the linear predictor. In XGBoost models, we included age with covariates in model 2 as predictive variables. We summarised model discrimination by calculating the AUC with 95% CIs.

To evaluate the clinical utility of the plasma protein-based prediction model, we assessed its ability to stratify participants according to their long-term risk of developing BPH. We dichotomised participants into Q1 (<median) and Q2 (≥median) groups based on the median value of the three protein levels. We visualised the cumulative incidence of BPH between these two groups using Kaplan-Meier survival curves, and tested the statistical significance of the difference in BPH-free survival with the log-rank test.

## RESULTS

### Participant baseline characteristics

We included 20 996 male participants with a median follow-up of 13.38 years, during which 2405 incident BPH cases were identified (**Table 1**). Participants with incident BPH were significantly older than those who remained BPH-free (*P* < 0.001). The BPH group also had a higher proportion of individuals with an annual family income<GBP 31 000 (*P* < 0.001) and a lower proportion with a college-level or above education (*P* < 0.001). In addition, males who developed BPH were more likely to be obese, smokers, and have a higher prevalence of using cholesterol-lowering drugs and blood pressure-lowering drugs (*P* < 0.001). The BPH group had slightly higher HbA1c levels (x̄ = 36.53; SD = 5.43) than the normal group (x̄ = 35.88; SD = 5.23) (*P* < 0.001), while the normal group had higher LDL cholesterol levels (x̄ = 3.47; SD = 0.85) than the BPH group (x̄ = 3.32; SD = 0.84) (*P* < 0.001).

**Table 1:** Baseline characteristics of participants*

	Normal, n = 18 591	BPH, n = 2405	Overall, n = 20 996	*P*-value
**Age in years, x̄ (SD)**	56.07 (8.38)	60.76 (6.50)	56.60 (8.32)	<0.001
**Family income (GBP)**				<0.001
<31 000	9649 (51.90)	1481 (61.58)	11 130 (53.01)	
≥31 000	8942 (48.10)	924 (38.42)	9866 (46.99)	
**Ethnicity**				0.061
White	17 249 (92.78)	2257 (93.85)	19 506 (92.90)	
Non-white	1342 (7.22)	148 (6.15)	1490 (7.10)	
**Educational levels**				<0.001
College or above	8583 (46.17)	998 (41.50)	9581 (45.63)	
High school or equivalent	6825 (36.71)	889 (36.96)	7714 (36.74)	
Less than high school	3183 (17.12)	518 (21.54)	3701 (17.63)	
**Smoking**				<0.001
Never	9091 (48.90)	1094 (45.49)	10 185 (48.51)	
Previous	7023 (37.78)	1060 (44.07)	8083 (38.50)	
Current	2477 (13.32)	251 (10.44)	2728 (12.99)	
**BMI (kg/m^2^)**				0.001
<25	4712 (25.35)	524 (21.79)	5236 (24.94)	
25–30	9269 (49.86)	1258 (52.31)	10 527 (50.14)	
>30	4610 (24.80)	623 (25.90)	5233 (24.92)	
**Alcohol consumption**				0.771
Never	569 (3.06)	76 (3.16)	645 (3.07)	
Previous	714 (3.84)	99 (4.12)	813 (3.87)	
Current	17 308 (93.10)	2230 (92.72)	19 538 (93.06)	
**Cholesterol-lowering drugs**				<0.001
No	14 350 (77.19)	1591 (66.15)	15 941 (75.92)	
Yes	4241 (22.81)	814 (33.85)	5055 (24.08)	
**Blood pressure-lowering drugs**				<0.001
No	14 078 (75.72)	1587 (65.99)	15 665 (74.61)	
Yes	4513 (24.28)	818 (34.01)	5331 (25.39)	
**Hba1c (mmol/mol), x̄ (SD)**	35.88 (5.23)	36.53 (5.43)	35.95 (5.25)	<0.001
**LDL (mmol/L), x̄ (SD)**	3.47 (0.85)	3.32 (0.84)	3.45 (0.85)	<0.001

BMI – body mass index, BPH – benign prostatic hyperplasia, LDL – low-density lipoprotein, SD – standard deviation, x̄ – mean

*Presented as n (%) unless specified otherwise.

### BPH-associated plasma proteins

**Figure 1:**
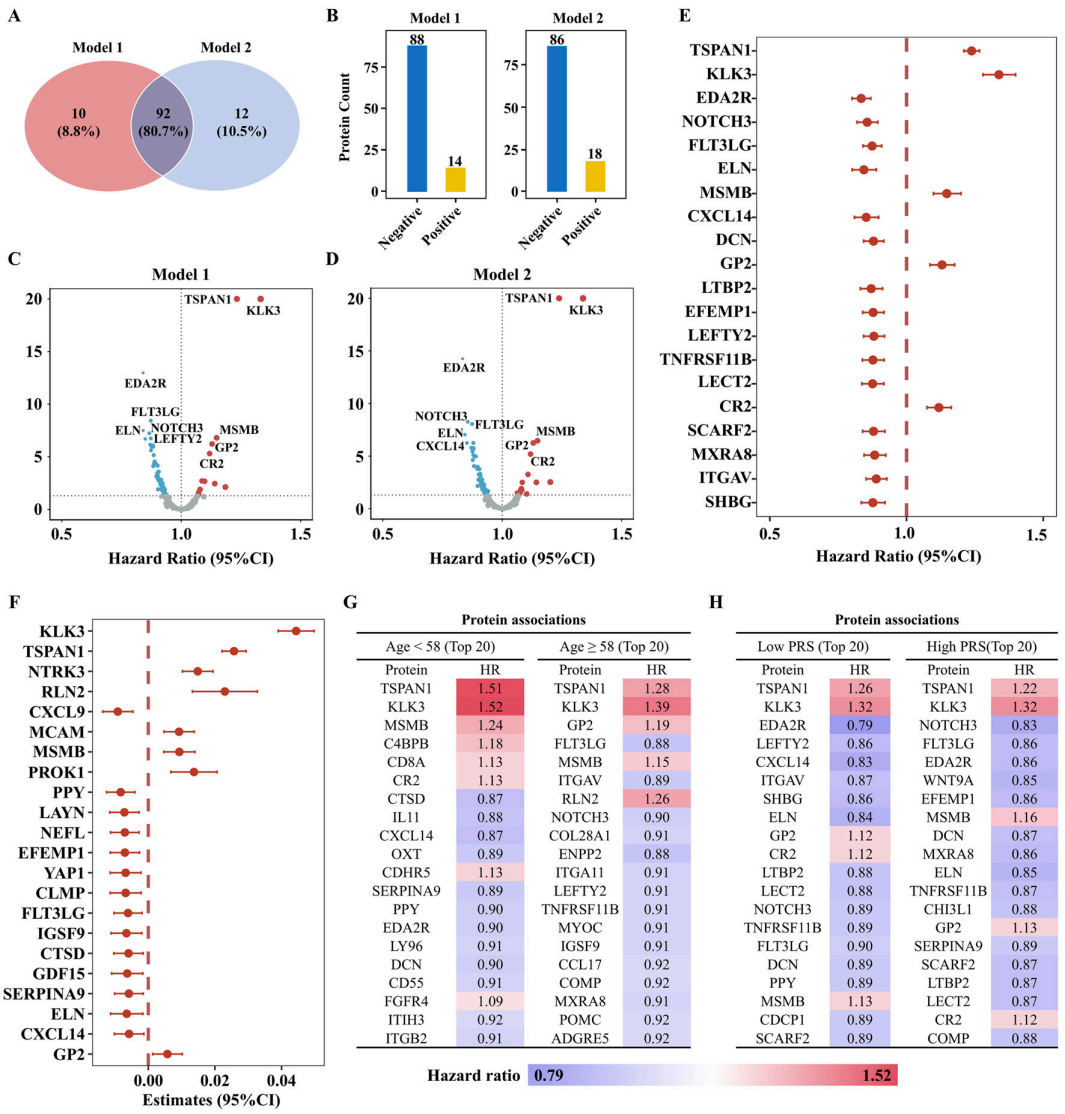
Identifying BPH-associated plasma proteins across different genetic risk strata. **Panel A**. Number of significantly associated proteins in models 1 and 2. **Panel B**. Number of positively and negatively associated proteins in models 1 and 2. **Panel C.** Volcano plots of the associated proteins in model 1. **Panel D.** Volcano plots of the associated proteins in model 2. **Panel E**. Top 20 proteins of the 92 significant proteins. **Panel F**. Significant proteins associated with the PRS of BPH. **Panel G**. Subgroup analysis by age. **Panel H.** Subgroup analysis by PRS groups. BPH – benign prostatic hyperplasia, CI – confidence interval, PRS – polygenic risk score.

We assessed the association between the plasma abundance of 2920 proteins and the risk of incident BPH using Cox proportional hazards regression. After adjusting for covariates in model 1, 102 plasma proteins were significantly associated with BPH risk (FDR<0.05). After adjusting for all covariates, 104 plasma proteins reached the FDR threshold of 0.05 (Tables S3 and S4 in the **Online Supplementary Document**). Among these, 92 proteins remained significant in both model 1 and model 2, with consistent effect estimate direction. Most significant proteins exhibited a negative association with BPH (**Figure 1**, Panels A and B). The top five proteins demonstrating the strongest positive associations with BPH remained unchanged in model 1 and model 2 (**Figure 1**, Panels C and D). In contrast, CXCL14 replaced LEFTY2 as one of the top five proteins negatively associated with BPH in the full model.

After adjusting for all covariates, the top 20 proteins remained out of the 92 significant proteins (**Figure 1**, Panel E). The top five positive proteins were TSPAN1 (HR = 1.24; 95% CI = 1.21–1.27; FDR = 1.69 × 10^−69^), KLK3 (HR = 1.34; 95% CI = 1.28–1.40; FDR = 1.48 × 10^−34^), MSMB (HR = 1.15; 95% CI = 1.10–1.20; FDR = 3.47 × 10^−7^), GP2 (HR = 1.13; 95% CI = 1.09–1.18; FDR = 5.58 × 10^−7^), and CR2 (HR = 1.12; 95% CI = 1.07–1.16; FDR = 6.43 × 10^−6^). The top five negative proteins were EDA2R (HR = 0.83; 95% CI = 0.80–0.87; FDR = 5.53 × 10^−15^), NOTCH3 (HR = 0.86; 95% CI = 0.82–0.90; FDR = 5.50 × 10^−9^), FLT3LG (HR = 0.87; 95% CI = 0.84–0.91; FDR = 8.78 × 10^−9^), ELN (HR = 0.84; 95% CI = 0.80–0.89; FDR = 9.06 × 10^−8^), and CXCL14 (HR = 0.85; 95% CI = 0.81–0.90; FDR = 5.58 × 10^−7^). Of the 92 significant proteins, 22 proteins showed significant association with PRS, with KLK3 showing the strongest association (**Figure 1**, Panel F). These findings reveal a distinct plasma proteomic signature associated with the development of BPH.

In the subgroup analyses, TSPAN1 and KLK3 showed consistent risk associations in both younger (<58 years) and older (≥58 years) age groups, as well as in both low and high PRS categories (**Figure 1**, Panels G and H). Other proteins, such as MSMB, GP2, and NOTCH3, showed stratum-specific variations in their HRs. However, interaction tests did not yield statistically significant evidence of effect modification by age or PRS for these proteins.

### Protein interactions, functional enrichment, and MR analysis

**Figure 2:**
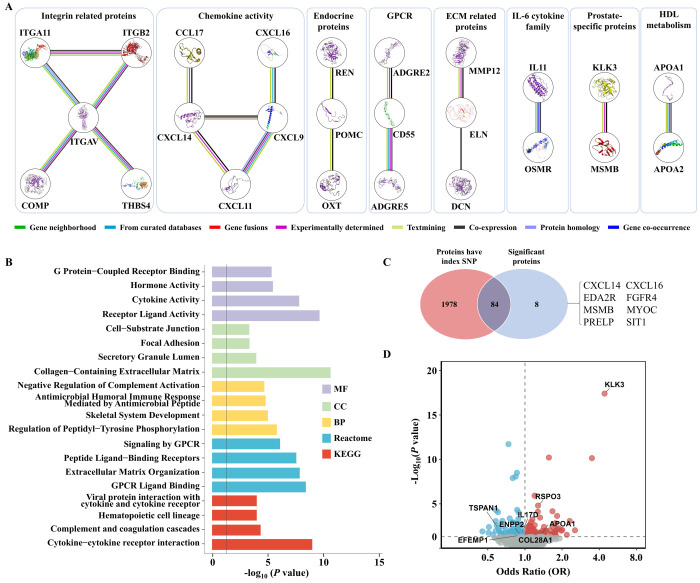
Enriched pathways and causal inference of identified proteins. **Panel A**. PPI network of the 92 significantly associated proteins. **Panel B**. Enrichment analysis of identified proteins. **Panel C**. Vene plot of significant proteins and those having an index SNP. **Panel D**. Volcano plot of the MR analysis results. PPI – protein-protein interaction, SNP – single-nucleotide polymorphism, MR – Mendelian randomisation.

To elucidate the functional relationships among the 92 BPH-associated plasma proteins, we constructed a PPI network. Of these, 22 showed connected interaction that was further separated by k-means clustering into eight functional clusters (**Figure 2**, Panel A). The functional clusters revealed a coordinated proteomic landscape that goes beyond the conventional hormone-centric aetiology of BPH, with a dominant role of immune-stromal crosstalk, inflammatory signalling, and tissue remodelling. Core clusters that drove this crosstalk include: chemokine signalling (*i.e.* CXCL16, CXCL14, CXCL9), that are pivotal for directing immune cell migration to the prostate microenvironment; integrin-mediated adhesion (*i.e.* ITGA11, ITGB2), that mediates leukocyte attachment and transmigration; extracellular matrix organisation and remodelling, including macrophage secreted metalloproteinase MMP12, that collectively regulate stromal stiffness and compliance; and G protein-coupled receptor ligand activity (*i.e.* ADGRE2, POMC), including receptors that are central to inflammatory and endocrine signalling; and IL-6 cytokine family members (*i.e.* OSMR, IL11), that are known drivers of local and systemic inflammation. In addition, the gold standard of prostate enlargement (KLK3, encoding PSA, and MSMB) were embedded in the clusters, adding more credibility to this proteomics work.

Functional enrichment analysis of 92-proteins further validated these observations and showed enrichment of cytokine activity, G protein-coupled receptor binding, focal adhesion, collagen-containing extracellular matrix and cytokine-cytokine receptor interaction, all non-androgenic pathways (**Figure 2**, Panel B). This pattern supports a pathophysiological model in which immune cell recruitment, chronic inflammation, and stromal remodelling act in concert, and independently of traditional hormonal drivers, to promote prostatic hyperplasia.

Finally, to assess potential causal relationships, we performed a two-sample cis-MR analysis. Of the 2062 proteins with suitable genetic instruments, 84 of 92 BPH-associated proteins had index cis-pQTLs to be adopted as instrumental variants for MR. MR analysis indicated that genetically predicted levels of eight proteins were nominally associated (*P* < 0.05) with BPH risk (**Figure 2**, Panels C and D). These included KLK3, RSPO3, IL17D, APOA1, COL28A1, ENPP2, EFEMP1, and TSPAN1.

### Assessment of protein importance and predictive performance

**Figure 3:**
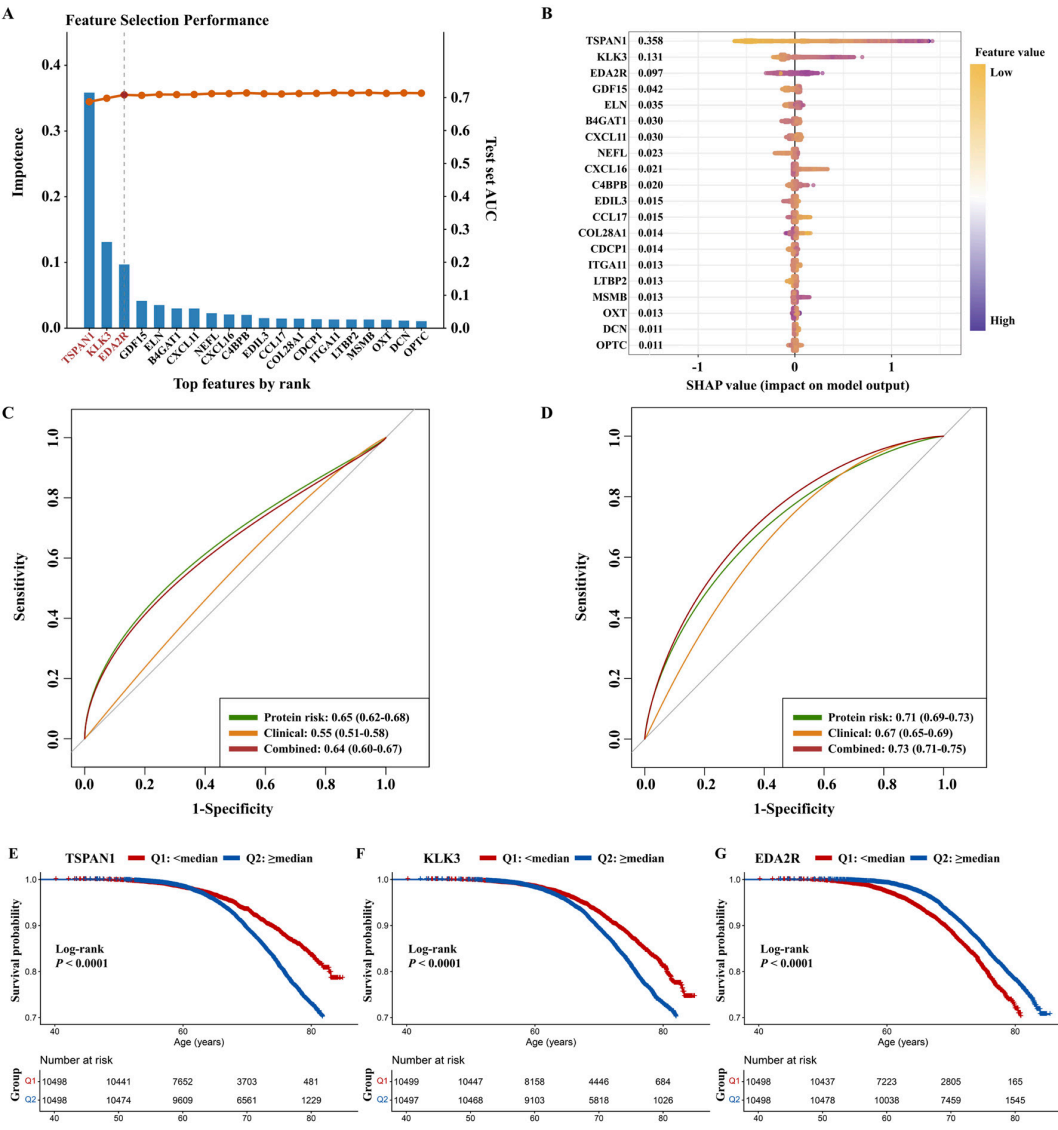
Assessment of protein importance and predictive performance. **Panel A**. Relative importance of the 92 significantly associated proteins with BPH. **Panel B**. The SHAP summary plot for the top 20 proteins. **Panel C.** Cox regression predictive accuracy of the identified three-protein panel (TSPAN1, KLK3, and EDA2R). **Panel D.** XGBoost Predictive accuracy of the identified three-protein panel (TSPAN1, KLK3, and EDA2R). **Panel E.** Kaplan-Meier survival curve by TSPAN1 level. **Panel F**. Kaplan-Meier survival curve by KLK3 level. **Panel G**. Kaplan-Meier survival curve by EDA2R level. SHAP – SHapley Additive exPlanations, XGBoost – Extreme Gradient Boosting.

Based on XGBoost, we subsequently evaluated the relative importance of the 92 significantly associated proteins with BPH. Through forward stepwise selection, we added proteins to the model in descending order of importance and calculated the corresponding AUC. The AUC rose steeply with the addition of the first three proteins. To interpret the contribution of each feature to individual predictions, we calculated SHA*P* values (**Figure 3**, Panels A and B). The top three absolute SHA*P* values for the proteins were TSPAN1 (x̄ = 0.358), KLK3 (x̄ = 0.131), and EDA2R (x̄ = 0.097).

We subsequently examined the predictive ability of the identified three-protein panel (TSPAN1, KLK3, and EDA2R) using two separate modelling strategies. First, we employed a Cox proportional hazards model. The model comprising only the three-protein panel had the highest AUC (AUC = 0.65; 95% CI = 0.62–0.68), followed by the combined model comprising both protein and clinical data (AUC = 0.64; 95% CI = 0.60–0.67), and the model comprising only the clinical covariates (AUC = 0.55; 95% CI = 0.51–0.58) had the lowest AUC (**Figure 3**, Panel C). Second, we used XGBoost to account for potential nonlinear relationships. In this analysis, the highest AUC was observed for the combined XGBoost model (AUC = 0.73; 95% CI = 0.71–0.75), followed by the model using only three-protein panel (AUC = 0.71; 95% CI = 0.69–0.73), and the model comprising only the clinical covariates (AUC = 0.67; 95% CI = 0.65–0.69) had the lowest AUC (**Figure 3**, Panel D). These results suggested that the clinical covariates provide limited additional predictive information. The model performance was largely driven by the plasma protein levels.

### Protein-based risk stratification of BPH onset

To assess the utility of the identified proteins for risk stratification, we adopted Kaplan-Meier survival analysis to evaluate the association between baseline plasma protein levels and the subsequent incidence of BPH. We stratified the participants into two groups based on the median plasma concentration of each protein: a lower-level group (Q1, <median) and a higher-level group (Q2, ≥median). Individuals with higher baseline levels of TSPAN1 had a significantly higher probability of developing BPH compared to those with lower levels (log-rank *P* < 0.0001) (**Figure 3**, Panel E). A similar pattern was observed for KLK3 (encoding PSA), where the Q2 group exhibited a significantly increased risk of BPH onset compared to the Q1 group (log-rank *P* < 0.0001). In contrast, the relationship between EDA2R and BPH risk was inverse (**Figure 3**, Panels F and G). Participants with higher baseline plasma levels of EDA2R demonstrated a significantly lower probability of developing BPH (log-rank *P* < 0.0001), suggesting a potential protective association. The divergent directions of association highlight the complexity of the proteomic landscape in BPH pathogenesis and underscore their collective utility for risk stratification.

## DISCUSSION

With this prospective cohort study of 20 996 male participants, we identified a distinct plasma proteomic signature for incident BPH, comprising 92 significantly associated proteins. The refined predictive model highlights a core three-protein signature – TSPAN1 and KLK3 as risk factors and EDA2R as a protective factor – with modest predictive performance. The underlying PPI network revealed a coordinated landscape extending beyond the conventional hormone-centric model, implicating immune-stromal crosstalk, inflammatory signalling, and tissue remodelling as central pathways in BPH aetiology.

The biological plausibility of the top three biomarkers reinforced the validity of our findings. KLK3 (encoding PSA) served as a critical positive control; its positive association with BPH aligns with its well-established role as a prostate epithelium-derived protein and the cornerstone of current prostate disease management [17,18]. Similarly, the significant association of MSMB, another major prostate-secreted protein [19], further validates the accuracy of our proteomic screen. The novel biomarkers TSPAN1 and EDA2R offer new mechanistic insights. TSPAN1 is a tetraspanin involved in cell adhesion, migration, and signal transduction [20,21]. In prostate pathologies, TSPAN1 is linked to cell proliferation and migration [22,23], suggesting its plasma levels reflect early prostatic dysregulation. Conversely, EDA2R, a tumour necrosis factor receptor involved in apoptosis and inflammation [24–26], exhibited a protective association, potentially indicating a role in controlling aberrant cellular growth in the ageing prostate.

The enrichment of immune pathways represents a significant shift from the traditional androgen-centric view of BPH. While androgens are established promoters of prostatic growth [1], emerging evidence suggests that androgens mainly promote BPH development after cellular damage and inflammation are generated, rather than onset [27]. Our data also support this paradigm. The identified chemokine signalling axis (*e.g.* CXCL16, CXCL14, CXCL9, CXCL11, CCL17) is instrumental in recruiting immune cells (*e.g.* T cells, macrophages, dendritic cells) to the prostate microenvironment [28]. Yan *et al.* reported CD8^+^ T cell and macrophage infiltration in the transitional zone of aged human prostate [29]. Joseph *et al.* also identified an influx of myeloid dendritic cells, T cells and B cells into the prostate, associated with BPH [30]. The recruited immune cells release growth factors and cytokines that directly stimulate stromal and epithelial proliferation [30,31]. This process is further sustained by enriched pathways such as integrin-mediated adhesion (facilitating leukocyte retention), extracellular matrix reorganisation (involving proteins like MMPs), and cytokine-cytokine receptor interactions (*e.g.* IL-6 family) [31]. Collectively, this pattern points to a self-perpetuating cycle of immune recruitment and tissue growth that may operate in parallel to, or even initiate, androgen-driven hyperplasia.

While functional enrichment analysis of the 92 associated proteins highlighted pathways related to inflammation and immune regulation, only 22 proteins formed a connected network at the highest confidence interaction threshold (STRING score >0.9). The remaining associated proteins were not incorporated into this core structure, suggesting that the identified proteomic signature is relatively heterogeneous and likely encompasses multiple distinct biological processes in addition to the cytokine-cytokine receptor axis. This is consistent with the complex, multi-aetiological nature of BPH. Moreover, plasma proteins are systemic markers, and enrichment of immune-inflammatory pathways does not necessarily indicate such alteration in the prostate. These associations could equally reflect broader metabolic or systemic inflammatory states rather than prostate-specific pathophysiological processes.

Clinically, the identification of a pre-symptomatic plasma proteomic signature holds promise, though its current performance requires cautious interpretation. Current prostate assessment relies heavily on symptom evaluation and prostate-specific markers like PSA [32] and non-specific molecules such as plasma metabolites [33] and neutrophil-to-lymphocyte ratio [34], with limited tools for early risk prediction. Our three-protein panel, which includes both prostate-specific (KLK3) and novel systemic (TSPAN1 and EDA2R) markers, provides a quantifiable, non-invasive method for early risk stratification. However, the three-protein panel achieved only moderate discrimination (AUC = 0.71) and adding clinical covariates did not significantly improve performance. This may indicate redundancy or that the proteins already capture the predictive information contained in covariates such as age. Therefore, these findings should be viewed as exploratory rather than evidence of ready clinical utility. Nevertheless, with further refinement, such a proteomic signature could eventually enable personalised monitoring schedules and timely intervention, potentially avoiding irreversible obstructive bladder dysfunction.

Our study has several strengths, including its large-scale prospective design, high-dimensional proteomic profiling, and multi-analytic framework integrating machine learning and genetic causality assessment. However, certain limitations need to be considered. First, while we identified a robust proteomic signature, the exact molecular mechanisms by which novel proteins like TSPAN1 influence prostate enlargement remain to be fully elucidated. Future research should prioritise functional validation in animal models and investigate whether therapeutic targeting of identified causal proteins can halt disease progression. Second, despite the promising performance of the identified proteomic signature, external validation in independent, multi-ethnic cohorts is essential to confirm generalisability and define clinical utility. Our findings should be viewed as hypothesis-generating for biomarker discovery rather than supporting immediate implementation or therapeutic targeting. In addition, defining incident BPH using International Statistical Classification of Diseases (tenth revision) code N40 from hospital inpatient records likely underestimates the true disease burden and introduces detection bias, as many cases are managed in outpatient or primary care settings without hospitalisation. Therefore, our outcome primarily captures clinically recognised or more severe BPH requiring inpatient care, rather than the full spectrum of incident disease in the community. Future studies incorporating primary care or community-based diagnostic codes are needed to capture milder or earlier-stage BPH. Finally, unmeasured confounders such as prostate volume, LUTS, PSA testing history, and prostate medication use were not available in the UKB, and residual confounding by these factors cannot be ruled out and may affect both association estimates and predictive performance. Future studies with more comprehensive phenotyping are needed to confirm our findings.

## CONCLUSIONS

With this large-scale prospective proteomic study, we identified a novel, three-protein plasma signature for incident BPH, highlighting TSPAN1 and KLK3 as key risk predictors and EDA2R as a protective factor. In addition, these identified proteins centred on immune-inflammatory and stromal remodelling pathways, expanding the etiological framework beyond androgen signalling. These findings provide a foundation for developing a non-invasive tool for early risk assessment and highlight novel therapeutic pathways of BPH.

## Additional material


Online Supplementary Document
