# Supplementary material for: Plasma proteomic signatures predict incident benign prostatic hyperplasia: a prospective cohort study of 20 996 men
Source: J Glob Health. 2026 Jun 12;16:04185. doi: 10.7189/jogh.16.04185 (PMC13261327; doi:10.7189/jogh.16.04185)
Supplement: Online Supplementary Document [file jogh-16-04185-s001.pdf]

Supplement to: Hao L, Yangchang Z, Li C, Jiuhong Y, Feng Q, Xianding W, Yang X. Plasma proteomic signatures predict incident benign prostatic hyperplasia: a prospective cohort study of 20 996 men. J Glob Health. 2026;16:04185.

Supplemental materials

1. **Table S1**：Adherence to JoGH's GRABDROP guidelines items (**Page 2**).
2. **Table S2**：SNPs used for individual PRS calculation (**Page 3-4**).
3. **Table S3**：Proteins significantly associated with BPH in Model 1 (**Page 5-7**).
4. **Table S4**：Proteins significantly associated with BPH in Model 2 (**Page 8-10**).
5. **Figure S1**：Flowchart of data cleansing (**Page 11**).

Table S1. Adherence to JoGH's GRABDROP guidelines items.

**JoGH guideline items**
1. Please list all papers published by each co-author in previous 3 years that were based on secondary analysis of a big data repository
Hao Li: None; Yangchang Zhang: None; Li Chen: None; Feng Qin: None; Xianding Wang: None.<br>Jiuhong Yuan: Evaluating the safety profile of α-1 blockers, 5α-reductase inhibitors, and PDE5I for BPH: a disproportionality analysis of real-world adverse events based on FDA adverse event reporting system (FAERS). *Aging Male*. 2026;29(1):2647019.<br>Yang Xiong: Insights into modifiable risk factors of erectile dysfunction, a wide-angled Mendelian Randomization study. *Journal of Advanced Research*. 2024; 58:149-161.
2. Please explain the key elements of your study design and the use of the available datasets that make your study an original scientific contribution
The originality of our study lies in the combination of a prospective proteome-wide association design, the systematic screening of 2,920 plasma proteins from the UK Biobank, and a focus on incident benign prostatic hyperplasia (BPH)—a condition where large-scale biomarker discovery has been largely absent. Unlike previous cross-sectional or small-scale studies, our work uses a prospective cohort to identify protein signatures associated with future BPH diagnosis, thereby minimising reverse causation. The UK Biobank provides an unprecedented opportunity to measure thousands of circulating proteins in a population-based sample, allowing us to move beyond non-specific markers such as PSA. We further strengthen the contribution by validating the discovered proteomic signatures, evaluating their predictive performance through machine learning, and investigating the underlying biological pathways. To our knowledge, this is the first large-scale prospective study that systematically screens the plasma proteome to predict incident BPH, offering a novel tool for early risk stratification and shifting the current reactive, symptom-based management toward a more personalised, pre-symptomatic approach.
3. Please list all publications that addressed similar research questions in the same dataset and indicate where you cited them in your paper
In the same dataset (UK Biobank), no manuscript was found with similar research questions.
4. Please explain how you addressed multiple testing through an appropriately rigorous statistical threshold and indicate this in the methods section
We used False Discovery Rate via Benjaminiand Hochberg method to address multiple testing. An FDR-adjusted P-value < 0.05 was considered significantly. This was indicated in the Methods section.
5. Please declare to what extent have AI chatbots been used in developing your paper and to which parts of the paper did they contribute
No AI chatbots was used during the manuscript preparation.

Table S2: SNPs used for individual PRS calculation

SNP	Effect Allele	Other Allele	*P*	BETA	SE	EAF
rs10788928	A	T	3.68E-08	0.0529	0.0096	0.3053
rs17467793	G	T	1.12E-08	-0.0508	0.0089	0.5351
rs4832620	T	C	1.02E-17	-0.1016	0.0119	0.8319
rs2556378	G	T	1.32E-20	-0.0988	0.0106	0.7801
rs2955117	A	G	1.90E-08	0.0536	0.0095	0.3120
rs7624084	C	T	2.26E-14	0.0679	0.0089	0.4486
rs61407096	G	A	2.21E-09	-0.0831	0.0139	0.1180
rs6830443	A	G	3.07E-09	-0.0533	0.0090	0.4285
rs6832544	T	G	2.02E-11	0.0654	0.0098	0.2914
rs27068	T	C	4.69E-28	-0.1179	0.0107	0.2234
rs10038396	G	T	1.47E-13	0.0697	0.0094	0.6680
rs147721431	T	C	1.13E-08	0.1989	0.0348	0.0155
rs6895915	C	T	1.00E-08	-0.0513	0.0089	0.5724
rs6594514	A	G	5.51E-16	-0.0856	0.0106	0.2319
rs35375078	A	T	5.39E-13	-0.1266	0.0175	0.0704
rs35868206	A	C	5.37E-09	-0.0600	0.0103	0.2475
rs35162296	T	C	1.23E-12	-0.1437	0.0202	0.0518
rs7742369	G	A	3.01E-12	0.0872	0.0125	0.1454
rs9655205	A	C	1.89E-17	-0.0912	0.0107	0.7785
rs757137	C	T	5.79E-10	-0.0606	0.0098	0.2929
rs2942219	G	A	3.92E-10	-0.0628	0.0100	0.2734
rs1386374	A	G	1.01E-12	-0.0745	0.0105	0.2383
rs111724742	G	T	5.84E-09	0.2284	0.0392	0.0130
rs10107982	C	T	2.20E-14	-0.0803	0.0105	0.2326
rs7842552	G	A	4.01E-08	0.0521	0.0095	0.3215
rs17057093	G	A	3.45E-08	-0.0988	0.0179	0.0671
rs7905955	G	A	2.59E-16	-0.0943	0.0115	0.8212
rs7094089	T	A	1.02E-08	0.0640	0.0112	0.8005
rs1265005	G	A	2.81E-14	-0.1588	0.0209	0.9530
rs61875074	C	A	1.72E-09	-0.0876	0.0146	0.1062
rs10886897	C	T	5.56E-22	0.0932	0.0097	0.2963
rs10510095	C	T	1.05E-18	-0.1417	0.0160	0.0870
rs11551933	C	T	1.17E-15	-0.1327	0.0166	0.0802
rs183263508	G	A	1.68E-09	-0.1587	0.0263	0.0326
rs2062525	C	T	1.43E-09	-0.0884	0.0146	0.1065
rs3118904	A	G	4.63E-20	0.0817	0.0089	0.4508
rs76781425	A	G	2.77E-08	0.1053	0.0190	0.0557
rs78220468	T	C	5.66E-09	0.0805	0.0138	0.1174
rs55908306	A	G	2.04E-08	0.0658	0.0117	0.1697
rs251730	A	T	3.42E-09	0.0525	0.0089	0.5127
rs73572277	G	A	1.74E-13	0.1015	0.0138	0.1150
rs12102689	G	A	1.11E-08	0.0518	0.0091	0.4032

rs141248788	T	C	2.46E-08	0.2734	0.0490	0.0086
rs11263761	G	A	1.12E-18	-0.0817	0.0093	0.3605
rs8097576	C	T	4.95E-16	-0.0726	0.0089	0.5607
rs11084596	C	T	1.90E-34	-0.1124	0.0092	0.3831
rs17632542	C	T	1.02E-19	-0.1618	0.0178	0.0682
rs139923107	T	C	1.49E-14	-0.1569	0.0204	0.0512
rs2836750	G	T	3.31E-10	-0.0559	0.0089	0.4858
rs17879961	G	A	3.74E-13	0.1833	0.0252	0.0304
rs186951204	G	A	2.69E-09	0.3207	0.0539	0.0063
rs62236881	A	G	4.02E-12	0.3412	0.0492	0.0075
rs5969782	C	G	1.08E-20	-0.0606	0.0065	0.3762
rs7065158	G	A	1.66E-22	-0.0681	0.0070	0.7255
rs13441059	A	G	1.94E-22	0.0638	0.0065	0.3545

SNP: Single nucleotide polymorphism; PRS: Polygenic risk score; SE: Standard error; EAF: Effect allele frequency.

Table S2: Proteins significantly associated with BPH in Model 1

Proteins	HR	LCI	UCI	*P*	FDR
TSPAN1	1.23	1.21	1.26	3.20E-71	9.33E-68
KLK3	1.33	1.27	1.39	1.11E-36	1.63E-33
EDA2R	0.84	0.81	0.88	1.10E-16	1.07E-13
FLT3LG	0.87	0.84	0.91	5.05E-12	3.69E-09
ELN	0.84	0.80	0.89	5.52E-11	3.22E-08
NOTCH3	0.87	0.83	0.90	1.20E-10	5.82E-08
MSMB	1.15	1.10	1.20	3.79E-10	1.58E-07
LEFTY2	0.87	0.84	0.91	4.96E-10	1.81E-07
CXCL14	0.85	0.81	0.89	6.20E-10	2.01E-07
GP2	1.13	1.09	1.18	2.06E-09	6.00E-07
LECT2	0.87	0.83	0.91	2.65E-09	7.03E-07
DCN	0.88	0.85	0.92	3.45E-09	8.39E-07
ITGAV	0.88	0.85	0.92	5.25E-09	1.10E-06
TNFRSF11B	0.88	0.84	0.92	5.30E-09	1.10E-06
LTBP2	0.87	0.83	0.92	1.23E-08	2.40E-06
CR2	1.12	1.08	1.16	2.70E-08	4.94E-06
EFEMP1	0.88	0.85	0.92	4.07E-08	7.00E-06
MXRA8	0.89	0.85	0.93	1.73E-07	2.80E-05
SCARF2	0.89	0.85	0.93	3.06E-07	4.70E-05
COMP	0.89	0.86	0.93	3.32E-07	4.84E-05
CRTAC1	0.90	0.86	0.94	4.92E-07	6.84E-05
SHBG	0.88	0.84	0.93	5.78E-07	7.67E-05
SERPINA9	0.91	0.87	0.94	2.05E-06	2.60E-04
CDCP1	0.90	0.86	0.94	3.01E-06	3.58E-04
WNT9A	0.90	0.86	0.94	3.06E-06	3.58E-04
IGSF9	0.90	0.86	0.94	6.43E-06	7.22E-04
IL17D	0.90	0.85	0.94	6.72E-06	7.27E-04
APOA1	0.90	0.86	0.94	1.08E-05	1.13E-03
CCDC80	0.91	0.87	0.95	1.61E-05	1.52E-03
CHI3L1	0.92	0.88	0.95	1.55E-05	1.52E-03
GDF15	0.90	0.86	0.95	1.60E-05	1.52E-03
LY96	0.91	0.88	0.95	1.68E-05	1.53E-03
MMP12	0.91	0.88	0.95	1.85E-05	1.64E-03
CD8A	1.09	1.05	1.13	2.28E-05	1.96E-03
C4BPB	1.10	1.05	1.15	2.55E-05	2.13E-03
NEFL	0.91	0.87	0.95	3.08E-05	2.46E-03
VEGFB	0.92	0.88	0.95	3.12E-05	2.46E-03
PLXNB2	0.92	0.88	0.96	3.84E-05	2.95E-03
RSPO1	0.92	0.88	0.95	4.25E-05	3.18E-03
COL28A1	0.92	0.89	0.96	4.47E-05	3.26E-03
CXCL11	0.91	0.88	0.95	5.05E-05	3.54E-03
PROK1	1.14	1.07	1.22	5.09E-05	3.54E-03

CCL27	0.92	0.88	0.96	6.32E-05	4.27E-03
IGFBPL1	0.92	0.88	0.96	6.43E-05	4.27E-03
ADGRE5	0.92	0.88	0.96	6.81E-05	4.42E-03
OPTC	0.91	0.87	0.96	6.97E-05	4.43E-03
MCAM	0.92	0.88	0.96	7.21E-05	4.48E-03
CCL17	0.92	0.89	0.96	1.15E-04	7.00E-03
CD55	0.92	0.88	0.96	1.21E-04	7.22E-03
RLN2	1.18	1.09	1.29	1.29E-04	7.52E-03
NTRK3	0.92	0.88	0.96	1.33E-04	7.61E-03
GIP	0.93	0.89	0.96	1.41E-04	7.93E-03
ITGB2	0.92	0.88	0.96	1.55E-04	8.56E-03
CTSD	0.92	0.88	0.96	1.96E-04	1.02E-02
PPY	0.93	0.89	0.96	1.94E-04	1.02E-02
RSPO3	0.92	0.88	0.96	1.91E-04	1.02E-02
CHGB	0.93	0.89	0.96	2.24E-04	1.15E-02
CD70	0.93	0.89	0.96	2.45E-04	1.23E-02
CST6	1.08	1.04	1.12	2.49E-04	1.23E-02
ENPP2	0.90	0.85	0.95	2.56E-04	1.23E-02
OXT	0.93	0.89	0.96	2.57E-04	1.23E-02
FGFR4	1.08	1.04	1.13	2.71E-04	1.27E-02
LIPF	0.93	0.89	0.97	2.78E-04	1.27E-02
PRELP	0.93	0.90	0.97	2.74E-04	1.27E-02
POSTN	0.93	0.89	0.97	2.88E-04	1.29E-02
SNCG	0.92	0.88	0.96	3.05E-04	1.34E-02
YAP1	0.92	0.88	0.96	3.08E-04	1.34E-02
DKK3	0.93	0.89	0.97	3.44E-04	1.48E-02
B4GAT1	0.93	0.89	0.97	3.49E-04	1.48E-02
CXCL16	0.92	0.89	0.97	3.75E-04	1.57E-02
CXCL9	0.92	0.88	0.96	3.81E-04	1.57E-02
MSR1	0.93	0.89	0.97	3.92E-04	1.59E-02
ASPN	0.93	0.89	0.97	4.44E-04	1.76E-02
ITGA11	0.93	0.89	0.97	4.45E-04	1.76E-02
FUT3_FUT5	0.93	0.89	0.97	5.00E-04	1.95E-02
POMC	0.93	0.89	0.97	5.13E-04	1.95E-02
THBS4	0.93	0.89	0.97	5.09E-04	1.95E-02
SIT1	1.08	1.03	1.12	5.22E-04	1.95E-02
MYOC	0.93	0.89	0.97	5.31E-04	1.96E-02
FGFBP2	0.93	0.89	0.97	6.75E-04	2.47E-02
IL11	0.93	0.89	0.97	7.71E-04	2.78E-02
MLN	0.93	0.89	0.97	8.07E-04	2.87E-02
AXL	1.07	1.03	1.12	8.26E-04	2.91E-02
ITGB1	0.93	0.89	0.97	8.81E-04	3.06E-02
LAYN	0.93	0.89	0.97	9.06E-04	3.11E-02
ADGRE2	0.93	0.89	0.97	9.70E-04	3.28E-02

APOA2	0.93	0.90	0.97	9.76E-04	3.28E-02
CLMP	0.93	0.89	0.97	1.00E-03	3.33E-02
ISM1	0.93	0.89	0.97	1.02E-03	3.35E-02
F3	0.93	0.89	0.97	1.07E-03	3.46E-02
CDHR5	1.07	1.03	1.12	1.18E-03	3.79E-02
GDF2	0.93	0.89	0.97	1.22E-03	3.87E-02
OSMR	0.93	0.89	0.97	1.35E-03	4.22E-02
CCN3	0.93	0.89	0.97	1.40E-03	4.34E-02
CNTN5	0.93	0.89	0.97	1.50E-03	4.62E-02
CCL13	0.94	0.90	0.97	1.54E-03	4.68E-02
CCN1	0.93	0.89	0.97	1.64E-03	4.81E-02
EDIL3	0.93	0.89	0.97	1.65E-03	4.81E-02
ITIH3	0.93	0.89	0.97	1.63E-03	4.81E-02
REN	0.94	0.90	0.98	1.60E-03	4.81E-02
CST5	0.94	0.90	0.98	1.69E-03	4.85E-02
KIAA0319	0.93	0.89	0.97	1.69E-03	4.85E-02
CLEC10A	0.94	0.90	0.98	1.89E-03	5.36E-02

LCI: lower confidence interval, UCI: upper confidence interval, FDR: false discovery rate.

Table S4: Proteins significantly associated with BPH in Model 2.

Proteins	HR	LCI	UCI	P	FDR
TSPAN1	1.24	1.21	1.27	5.79E-73	1.69E-69
KLK3	1.34	1.28	1.40	1.01E-37	1.48E-34
EDA2R	0.83	0.80	0.87	5.68E-18	5.53E-15
NOTCH3	0.86	0.82	0.90	7.53E-12	5.50E-09
FLT3LG	0.87	0.84	0.91	1.50E-11	8.78E-09
ELN	0.84	0.80	0.89	1.86E-10	9.06E-08
MSMB	1.15	1.10	1.20	8.33E-10	3.47E-07
CXCL14	0.85	0.81	0.90	1.72E-09	5.58E-07
DCN	0.88	0.84	0.92	1.75E-09	5.58E-07
GP2	1.13	1.09	1.18	1.91E-09	5.58E-07
LTBP2	0.87	0.83	0.91	6.03E-09	1.60E-06
EFEMP1	0.88	0.84	0.92	7.12E-09	1.73E-06
LEFTY2	0.88	0.84	0.92	8.22E-09	1.85E-06
TNFRSF11B	0.88	0.84	0.92	1.26E-08	2.63E-06
LECT2	0.88	0.84	0.92	1.87E-08	3.64E-06
CR2	1.12	1.07	1.16	3.52E-08	6.43E-06
SCARF2	0.88	0.84	0.92	4.68E-08	8.04E-06
MXRA8	0.88	0.84	0.92	6.29E-08	1.02E-05
ITGAV	0.89	0.85	0.93	6.97E-08	1.07E-05
SHBG	0.88	0.83	0.92	1.61E-07	2.35E-05
GDF15	0.88	0.84	0.93	6.37E-07	8.86E-05
COMP	0.90	0.86	0.94	7.76E-07	1.03E-04
WNT9A	0.90	0.86	0.94	1.27E-06	1.62E-04
CRTAC1	0.90	0.87	0.94	1.43E-06	1.74E-04
SERPINA9	0.91	0.87	0.95	4.29E-06	5.01E-04
C4BPB	1.11	1.06	1.16	4.92E-06	5.46E-04
CDCP1	0.90	0.86	0.94	5.05E-06	5.46E-04
ADGRE5	0.91	0.87	0.95	8.95E-06	9.33E-04
MMP12	0.91	0.87	0.95	1.49E-05	1.50E-03
LY96	0.91	0.88	0.95	1.76E-05	1.71E-03
CHI3L1	0.92	0.88	0.95	1.94E-05	1.77E-03
IL17D	0.90	0.86	0.95	1.89E-05	1.77E-03
NEFL	0.91	0.87	0.95	2.29E-05	2.02E-03
PLXNB2	0.91	0.88	0.95	2.55E-05	2.19E-03
CCDC80	0.91	0.87	0.95	3.21E-05	2.68E-03
RLN2	1.20	1.10	1.31	3.62E-05	2.94E-03
CD8A	1.08	1.04	1.13	4.07E-05	3.13E-03
PROK1	1.14	1.07	1.22	4.04E-05	3.13E-03
VEGFB	0.92	0.88	0.96	5.14E-05	3.85E-03
OPTC	0.91	0.87	0.95	5.50E-05	4.02E-03
COL28A1	0.92	0.89	0.96	7.24E-05	4.93E-03
GIP	0.92	0.89	0.96	7.51E-05	4.93E-03

IGFBPL1	0.92	0.88	0.96	7.67E-05	4.93E-03
IGSF9	0.91	0.87	0.95	7.74E-05	4.93E-03
MCAM	0.92	0.88	0.96	7.17E-05	4.93E-03
RSPO1	0.92	0.88	0.96	7.76E-05	4.93E-03
APOA1	0.91	0.87	0.96	9.39E-05	5.83E-03
CD55	0.92	0.88	0.96	1.05E-04	6.33E-03
CXCL11	0.92	0.88	0.96	1.06E-04	6.33E-03
ENPP2	0.90	0.85	0.95	1.16E-04	6.77E-03
RSPO3	0.92	0.88	0.96	1.33E-04	7.63E-03
ITGB2	0.92	0.88	0.96	1.38E-04	7.73E-03
CHGB	0.92	0.89	0.96	1.74E-04	9.49E-03
LAYN	0.92	0.88	0.96	1.76E-04	9.49E-03
DKK3	0.92	0.88	0.96	1.79E-04	9.51E-03
ADGRE2	0.92	0.88	0.96	2.06E-04	1.08E-02
CXCL9	0.92	0.88	0.96	2.13E-04	1.09E-02
REN	0.92	0.88	0.96	2.33E-04	1.17E-02
FGFR4	1.08	1.04	1.13	2.42E-04	1.20E-02
PPY	0.93	0.89	0.97	2.95E-04	1.43E-02
YAP1	0.92	0.88	0.96	3.00E-04	1.43E-02
ITIH3	0.92	0.88	0.96	3.08E-04	1.45E-02
CST6	1.08	1.03	1.12	3.57E-04	1.66E-02
CDHR5	1.08	1.04	1.13	3.69E-04	1.69E-02
CD70	0.93	0.89	0.97	4.14E-04	1.82E-02
FUT3_FUT5	0.93	0.89	0.97	4.05E-04	1.82E-02
NTRK3	0.92	0.88	0.97	4.25E-04	1.82E-02
POSTN	0.93	0.89	0.97	4.42E-04	1.82E-02
PRELP	0.93	0.90	0.97	4.17E-04	1.82E-02
SNCG	0.92	0.88	0.97	4.31E-04	1.82E-02
TCTN3	1.08	1.03	1.12	4.36E-04	1.82E-02
CTSD	0.92	0.89	0.97	4.49E-04	1.82E-02
CD27	0.93	0.89	0.97	4.95E-04	1.98E-02
APOA2	0.93	0.89	0.97	5.08E-04	2.00E-02
ADGRG1	0.94	0.91	0.97	5.51E-04	2.14E-02
CLMP	0.92	0.88	0.97	6.04E-04	2.32E-02
ITGA11	0.93	0.89	0.97	6.15E-04	2.33E-02
LIPF	0.93	0.89	0.97	6.30E-04	2.33E-02
MYOC	0.93	0.89	0.97	6.28E-04	2.33E-02
THBS4	0.93	0.89	0.97	6.45E-04	2.36E-02
SIT1	1.08	1.03	1.12	7.61E-04	2.74E-02
SSC4D	1.08	1.03	1.12	7.68E-04	2.74E-02
CCL17	0.93	0.89	0.97	7.84E-04	2.76E-02
POMC	0.93	0.89	0.97	8.95E-04	3.11E-02
CCN3	0.93	0.89	0.97	9.11E-04	3.13E-02
F11	1.06	1.03	1.10	9.47E-04	3.22E-02

CCL27	0.93	0.89	0.97	1.02E-03	3.32E-02
F3	0.93	0.89	0.97	1.00E-03	3.32E-02
IL11	0.93	0.89	0.97	1.02E-03	3.32E-02
MSR1	0.93	0.89	0.97	1.00E-03	3.32E-02
OXT	0.93	0.89	0.97	1.10E-03	3.52E-02
INSL3	1.10	1.04	1.17	1.24E-03	3.93E-02
CA9	0.94	0.90	0.97	1.30E-03	4.05E-02
CXCL16	0.93	0.89	0.97	1.30E-03	4.05E-02
IL5RA	0.94	0.90	0.97	1.35E-03	4.15E-02
EDIL3	0.93	0.89	0.97	1.40E-03	4.26E-02
OGN	0.93	0.90	0.97	1.44E-03	4.35E-02
KIAA0319	0.93	0.89	0.97	1.46E-03	4.35E-02
PLTP	0.94	0.90	0.98	1.51E-03	4.46E-02
B4GAT1	0.93	0.89	0.97	1.66E-03	4.86E-02
AOC3	0.93	0.89	0.97	1.73E-03	4.90E-02
CNTN5	0.93	0.89	0.97	1.71E-03	4.90E-02
OSMR	0.93	0.89	0.97	1.73E-03	4.90E-02
DSG4	1.07	1.03	1.12	1.77E-03	4.96E-02

LCI: lower confidence interval, UCI: upper confidence interval, FDR: false discovery rate.

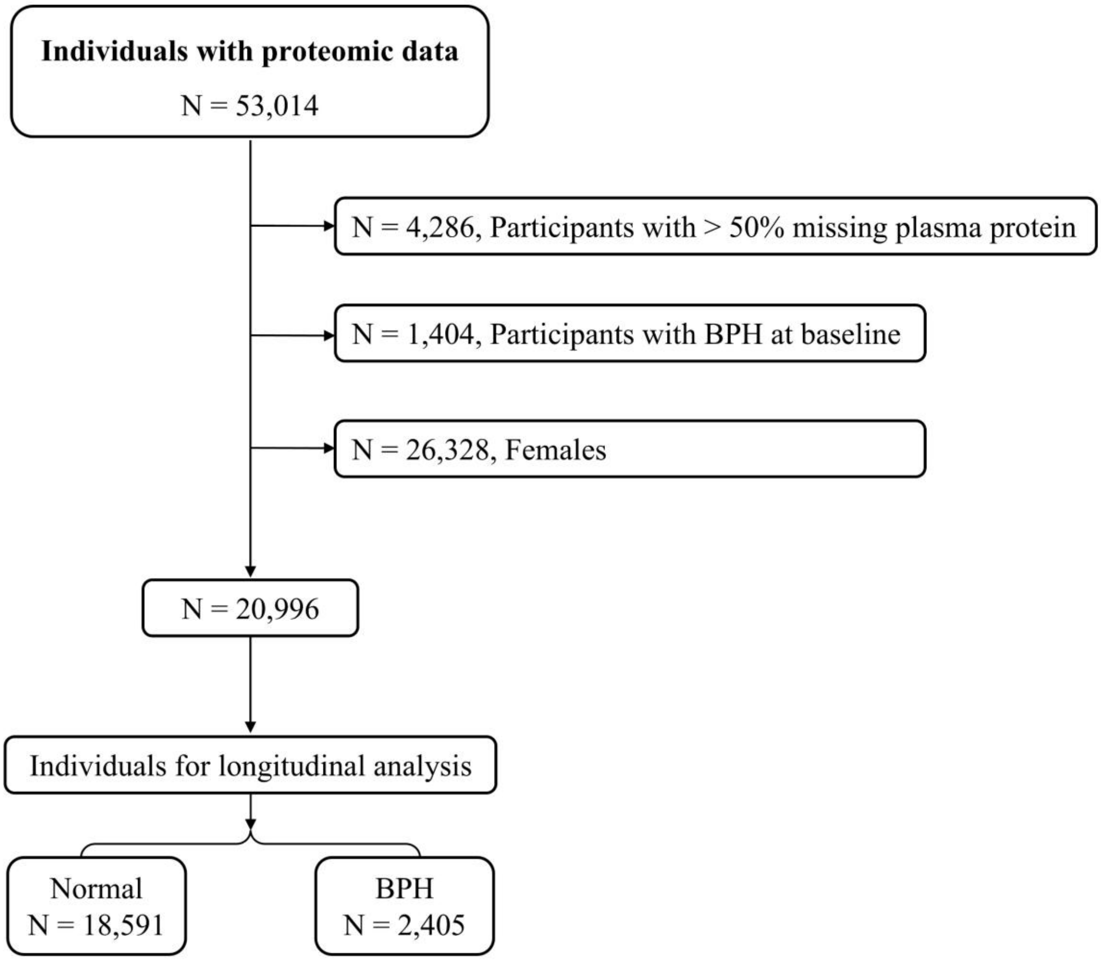

Figure S1: Flowchart of data cleansing.
